# Maternal Nutritional Environment and the Development of the Melanocortin System

**DOI:** 10.1002/cph4.70020

**Published:** 2025-06-06

**Authors:** Marina Galleazzo Martins, Alfonso Abizaid

**Affiliations:** ^1^ Department of Neuroscience Carleton University Ottawa Ontario Canada

**Keywords:** diabetes, energy balance, hypothalamus, obesity, offspring

## Abstract

The maternal nutritional and/or metabolic environment is crucial for future offspring health outcomes, and impairments during critical periods of development can alter the development of brain circuits that regulate energy balance, predisposing individuals to metabolic disorders throughout life. Epigenetic changes, changes in cell number and/or organ structure, and cellular metabolic differentiation could be some of the fetal adaptations leading to the development of metabolic disorders later in life. Here, we review animal models showing that the nutritional environment to which the offspring are exposed during their perinatal life can influence the development of the hypothalamic melanocortin system, promoting increased feeding and fat deposition. Following maternal undernutrition, the development of obesity in the offspring may be related to decreased POMC neuronal function since birth. Similarly, maternal diabetes and obesity also induce hypothalamic changes that result in an imbalance in AgRP/NPY and POMC expression during adulthood. Widespread impairments in brain development may also induce a global downregulation of the melanocortin system. Furthermore, animal models highlight that the time and type of exposure are key to the offspring outcomes, as are their sex and age. Possible sex‐specific differences remain unclear, as most studies have evaluated only the male offspring, despite females having an increased risk of developing obesity and gestational diabetes during their pregnancy, which imposes a transgenerational effect of metabolic disorders. Studies aiming at evaluating the long‐term effects of the maternal nutritional environment in both males and females could help delineate how the susceptibility to metabolic disorders development worsens over time.

## Graphical abstract

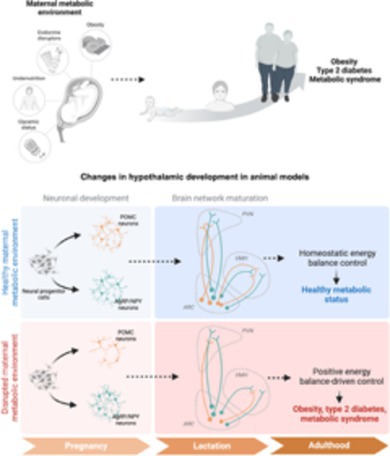

Obesity and metabolic disorders are directly related to most, if not all, chronic diseases and worsen health outcomes in human populations despite treatment for these diseases. Indeed, obesity is closely associated with most chronic disorders, including Type II diabetes, cardiovascular disease, renal disease, cancer, and mental health disorders (Stoops and Dar 2023). The factors associated with the development of obesity and metabolic disorders are, however, complex and involve a combination of genetic and lifestyle factors that confer vulnerability. A substantial amount of evidence suggests that the brain circuits regulating energy balance have critical periods of development and that changes in the maternal nutritional environment or metabolic state during these periods can alter the development of these circuits to be more vulnerable for the development of obesity and metabolic disorders later in life (see Figure 1).

**FIGURE 1:**
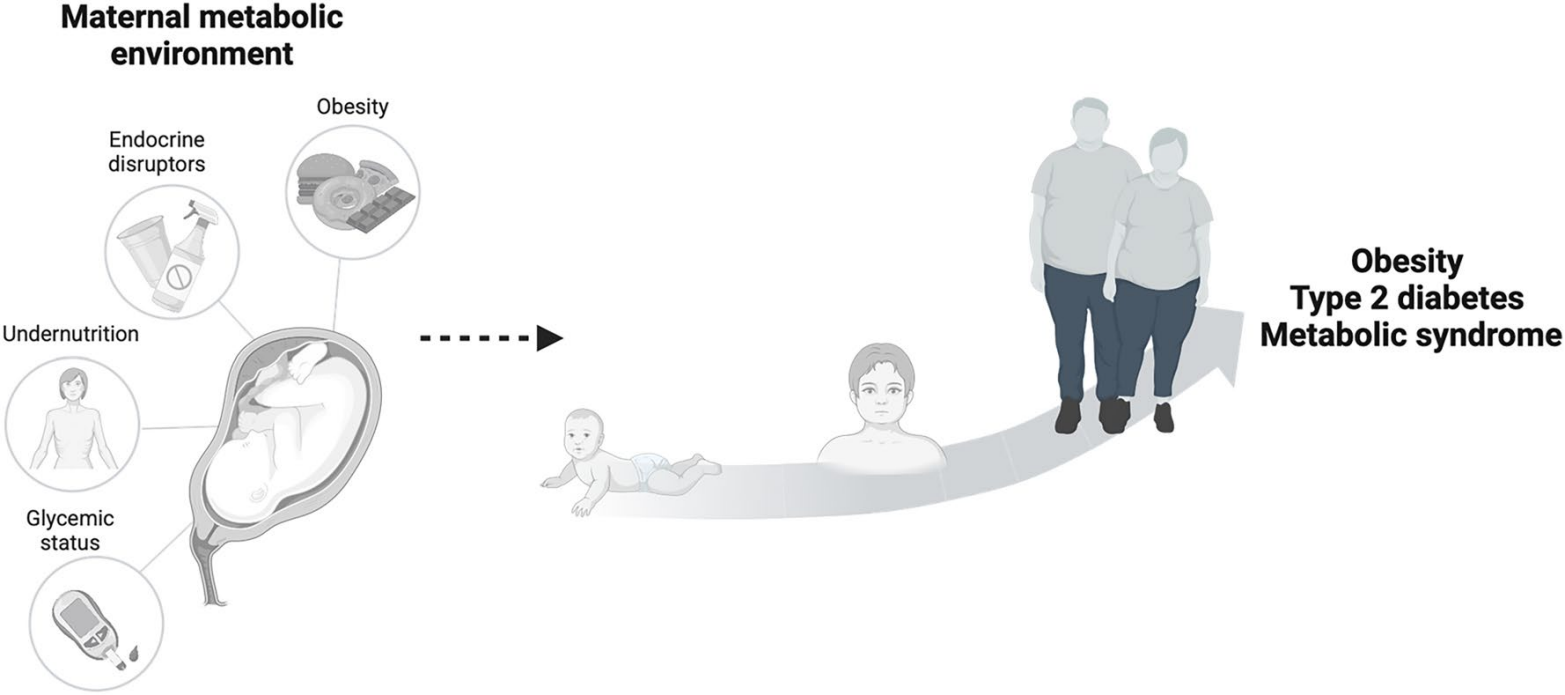
Maternal programming of metabolic disorders. The maternal metabolic environment is related to an increased risk of developing obesity, Type 2 diabetes, and metabolic syndrome in the offspring throughout life (Created in BioRender, https://BioRender.com/71h2bvr).

## 1 Maternal Nutrition and Infant Development

Maternal nutrition is an important determinant of fetal development and future health outcomes (Marshall et al. 2022). Fetal development depends on the nutrient supply transported from the mother to the offspring via the placenta (Jain et al. 2022), which includes adequate glucose, lipids, and amino acids levels (Larqué et al. 2013). Therefore, the maternal nutritional environment can affect the amount and quality of nutrients received by the fetus and change its development.

Both inadequate and excessive maternal nutrition during pregnancy and lactation are related to poor maternal and fetal outcomes, including short‐ and long‐term maternal metabolic disorders, obstetric complications, preterm delivery, infant mortality, and the development of metabolic disorders and noncommunicable diseases throughout life (Ramakrishnan et al. 2012; Fleming et al. 2018). Most importantly, fetal adaptations to an impaired maternal nutritional environment could underlie the development of metabolic disorders later in life (Tarrade et al. 2015). The mechanisms through which maternal nutritional status affects offspring's long‐term health include epigenetic changes, alterations in cell number and/or organ structure, and cellular metabolic differentiation (Koletzko et al. 2011).

Excessive maternal nutrient supply, as seen in obesity and gestational diabetes, leads to an increased risk of macrosomia in the offspring, accompanied by increased fat deposition and increased risk for developing obesity and Type 2 diabetes (Parsons et al. 2001; Catalano et al. 2003; Gillman et al. 2003). Interestingly, impaired nutrient supply, such as maternal undernutrition, also leads to poor metabolic outcomes in the offspring. Low birth weight and restricted fetal growth are a risk factor for increased fat deposition in adulthood and the development of metabolic disorders (Roseboom et al. 2006). Notably, offspring birth weight is a strong predictor of future outcomes (Fernandez‐Twinn et al. 2019), and both low and high birth weight are associated with the increased risk of obesity and metabolic syndrome later in life, and their mechanisms may be shared (Remmers and Delemarre‐van De Waal 2011).

Animal models have shown that maternal nutrition during pregnancy can alter placental structure and function, fetal growth, and offspring metabolism and energy balance (Remmers and Delemarre‐van De Waal 2011; Schoonejans and Ozanne 2021), including changes in body weight and composition and energy intake and expenditure. Ultimately, the nutritional environment to which the offspring is exposed during its perinatal life can influence hypothalamic development and the systems that regulate food intake and energy balance (Zeltser 2018), such as the melanocortin system.

Importantly, maternal nutritional environment and subsequent development of offspring can also be impacted by exposure to endocrine disruptors, chemical compound pollutants that are released into the environment and that have chemical properties that can mimic those of metabolic hormones and thus alter the development of the brain networks associated with metabolic regulation. Indeed, compounds used in fertilizers, plasticizers, and in the food industry in general (animal antibiotics and hormonal treatments) also impact the development of brain systems important for energy balance regulation (Heindel et al. 2022).

## 2 Melanocortin System

Energy balance is maintained by complex networks that interact to promote homeostasis. Arguably, given the importance of sustaining the energy requirements of life, it is not surprising that these systems encompass much of the brain (for review see Berthoud et al. 2020). Nevertheless, the hypothalamic melanocortin system is critical for energy regulation (Yeo et al. 2021). Indeed, cells within this system can monitor changes in peripheral nutrient signals like glucose or fatty acids, as well as changes in hormones that regulate feeding and metabolism. Importantly, mutations that alter components of the melanocortin system lead to morbid obesity or anorexia (Kobayashi et al. 2002; Mergen et al. 2001; Rajcsanyi et al. 2024; Vink et al. 2001).

The melanocortin system is common to vertebrates, including humans and rodents. The primary sensory cells within this system are peptidergic cells located in the hypothalamic arcuate nucleus (ARC). A set of these cells produces the proopiomelanocortin (POMC) gene that encodes the precursor for different peptides that function both as neuropeptides and hormones, including adrenocorticotropin (ACTH), α‐, β‐, and γ‐melanocyte‐stimulating hormone (MSH), and β‐endorphin. Critically, α‐MSH secreted from ARC POMC neurons is a potent appetite suppressant and increases energy expenditure through binding to G protein‐coupled melanocortin receptors (MC3R and MC4R), expressed throughout the hypothalamus, brainstem and a number of limbic regions (Mountjoy et al. 1994; Kishi et al. 2003). A second set of cells within the ARC produce the agouti‐related peptide (AgRP) and neuropeptide Y (NPY), both peptides that increase appetite and decrease energy expenditure (Cone 2005). AgRP is considered part of the melanocortin system since it binds to the same MC3R and MC4R to oppose the effects of α‐MSH (Cone 2005). Indeed, fasting decreases POMC expression and increases AgRP expression (Ahima et al. 1999; Bertile et al. 2003; Brady et al. 1990; Korner et al. 2001; Hagan et al. 1999; Van Dijk et al. 1999), whereas satiety signals like leptin increase POMC expression and α‐MSH release (Elias et al. 1998; Cowley et al. 2001) while decreasing AGRP expression and release. The role of the melanocortin system in energy balance control is also shown by monogenic disorders, with mutations in the *Mc4r* gene being the most common monogenic type of obesity (O'Rahilly et al. 2003).

The melanocortin system and its hypothalamic outputs can modulate energy balance in several ways, including activating motor neurons to initiate/terminate meals, promoting hormonal secretion, and changing autonomic tuning (Remmers and Delemarre‐van De Waal 2011). In addition to its effects on energy balance control, the melanocortin system has also been implicated in modulating cardiovascular and sexual physiology, all of which could also be affected by detrimental maternal nutritional status.

## 3 Development of Melanocortin System

Our understanding of the melanocortin system development comes particularly from animal models, specifically mice and rats. In rodents, the hypothalamus begins to develop on embryonic day (ED) 11 (Shimada and Nakamura 1973), with neurons expressing POMC and AgRP/NPY between ED 11 and 13 (Padilla et al. 2010). The expression of *Pomc* mRNA has been detected as early as ED 10, peaking at around weaning (Ahima and Hileman 2000). The mRNA for *Npy* is first detected on ED 14 (Padilla et al. 2010) and its expression reaches adult levels around birth (Singer et al. 2000). The developmental pattern of *Agrp* mRNA expression is similar to that of NPY (Grove et al. 2003; Nilsson et al. 2005). On ED 12, *Mc4r* mRNA is already detected in rodents, peaking on ED 16 (Mountjoy and Wild 1998), with some studies suggesting that this receptor is involved in hypothalamic development. Interestingly, POMC neurons during development can differentiate into other neuronal cells, such as NPY (Padilla et al. 2010; MacKay and Abizaid 2014). POMC and AgRP projections from the Arc to other hypothalamic nuclei, however, develop only after birth in rodents. Axons from AgRP and POMC neurons develop between Postnatal Day (PND) 7 and 14 (Grove et al. 2003; Nilsson et al. 2005), and peak between PND 8 and 10 in mice (Bouret et al. 2004a). Those projections reach their adult pattern on PND 18 (Bouret et al. 2004a), although mature synapses are seen only during puberty (Melnick et al. 2007).

In primates, hypothalamic development starts around Weeks 9–10 of pregnancy, and in humans, the structure of all major nuclei is already similar to adulthood by the 34th week of gestation (Koutcherov et al. 2002). However, one of the last neuronal cell types to develop in the human fetal brain is the NPY neurons of the ARC, with the earliest neurons appearing around the 21st week of pregnancy (Koutcherov et al. 2002).

Several factors regulate melanocortin system development, and these include genetic, molecular, and cellular factors, hormonal levels, and exposure to environmental signals including pollutants and drug compounds that mimic the effects of hormones that are key to the development of the hypothalamus. Early development is genetically driven by programmed expression of transcription factors and axon guidance molecules that orchestrate the development of hypothalamic networks like those comprising the melanocortin system neurons (Bouret 2022). Thus, changes in one or multiple of these molecular signals could lead to impairments in hypothalamic development that could have long‐lasting consequences for the organism (MacKay and Abizaid 2014).

For instance, several homeobox and basic helix–loop–helix (bHLH) transcription factors mediate the development of POMC neurons. While the retinal and anterior neural fold homeobox (Rax) is necessary for POMC expression (Lu et al. 2013), other transcription factors are related to a decrease in the number of POMC neurons during intrauterine development that may last until adulthood, such as the bone morphogenetic protein receptor 1A (Bmpr1A) (Peng et al. 2012), expressed in Olig1 progenitors (Samanta et al. 2007), neurogenin 3 (Ngn3) (Pelling et al. 2011), and Mash1 (McNay et al. 2006). The disrupted expression of Ngn3 in Nkx2.1 expressing cells leads to few POMC neurons throughout life, ultimately leading to hyperphagia and early onset of obesity (Anthwal et al. 2013).

Although the development of neuronal cell bodies is completed during intrauterine development, their connections to other regions only reach maturity during the postnatal period in rodents (Zeltser 2018), indicating that maternal metabolic disruptions during those two periods may impact melanocortin system structure and function. The postnatal development of the melanocortin system is regulated by changes in metabolic hormones in the first weeks of life, including changes in leptin and ghrelin, which show a peak in plasma concentrations early in life (Ahima et al. 1998; Bouret et al. 2004b; Steculorum et al. 2015). Between PND 10 and 16, the leptin surge promotes the development of axons from the ARC to the PVN in mice and rats (Bouret et al. 2004b; Kamitakahara et al. 2018) and is essential for the projections' density to reach adulthood levels (Bouret et al. 2004b). Ghrelin, on the other hand, has an inhibitory effect on axons' development in postnatal life. Between PND 6 and 14, circulating ghrelin levels increase, leading to a reduction in neurite extension (Steculorum et al. 2015). Blockade of ghrelin action during this period increases α‐MSH and AgRP fiber density in the PVN, DMH, and LHA, ultimately resulting in increased body weight gain and hyperglycemia (Steculorum et al. 2015). Importantly, ghrelin signaling during postnatal development inhibits leptin's neurotrophic effects (Steculorum et al. 2015), showing how both hormones interact to orchestrate those projections' development. Furthermore, the development of the melanocortin system is also affected by other hormones in early life, such as the growth hormone (Wasinski et al. 2020), glucagon‐like peptide 1, and amylin (Bouret 2022), which could all mediate how environmental signals as the maternal nutritional status are conveyed to the developing offspring (Dearden and Ozanne 2015).

## 4 Animal Models of Impaired Maternal Nutritional Environment

The idea that the maternal metabolic state may program offspring metabolism has come in part from human epidemiological studies showing that maternal food shortages or metabolic disorders are associated with vulnerability to develop metabolic disorders in the offspring (Roseboom et al. 2006; Lawlor et al. 2011; Catalano and deMouzon 2015; Perng et al. 2020; Lumey et al. 2011). Animal models of maternal exposure to metabolic challenges have been critical in understanding the mechanisms that render offspring more vulnerable to metabolic disorders, including those that alter the development of the melanocortin system and its function throughout life. These models include the development of maternal hyperglycemia, food restriction, access to high‐fat diets, maternal obesity, and maternal exposure to endocrine disruptors. Data from sources reviewed in this paper are summarized in Table 1 and described below.

**TABLE 1:** Animal models of maternal undernutrition and the development of the melanocortin system.

Species	Maternal intervention	Duration	Offspring	Age	Outcomes	a‐MSH	Receptors	AgRP/NPY	References
	Diet composition		Sex		POMC				
Mouse | CD‐1	50% protein restriction	PD 0 to PD 12	—	ED 12	↑ mRNA expression	x	x	↑ NPY mRNA expression; = AgRP mRNA expression	Terroni et al. (2005)
Rat | Sprague–Dawley	70% caloric restriction	PD 1–21	—	ED 12, 16 and 21	↓ mRNA expression on E12, E16 and E21	x	x	↑ NPY mRNA expression on E16 and E21; ↑ AgRP mRNA expression on E12, E16 and E21	Caminos et al. (2008)
Rat | Sprague–Dawley	50% caloric restriction	PD 10 to PD 21	Female	ED 18	↓ neurons in the ARC on E18; ↓protein and mRNA expression	x	x	x	Zhang et al. (2022)
Sheep	20% caloric restriction	PD −60 to 30	—	ED 131 and 135	= mRNA expression	x	x	= NPY mRNA expression	Stevens et al. (2010), Begum et al. (2012)
Sheep	30% caloric restriction	Pregnancy	Male and female	ED 130	= mRNA expression	x	x	↑ in NPY and AgRP mRNA expression	Adam et al. (2015)
Baboon	30% caloric restriction	PD 0 to birth	Male and female	ED 165	↓ neurons in the ARC	x	x	↑ NPY neurons in the ARC	Li et al. (2013)
Rat | Sprague–Dawley	50% caloric restriction	PD 10 to PD 21	Female	PND 1	↓neurons in the ARC; ↓protein and mRNA expression	x	x	x	Zhang et al. (2022)
Pig	50% caloric restriction	Last 2/3 of pregnancy	Male and female	PND 1	↓ mRNA expression in females	x	= MC4R mRNA expression	= NPY and AgRP mRNA expression	Óvilo et al. (2014)
Rat | Sprague–Dawley	50% caloric restriction	PD 10 to PD 21	Female	PND 5	↓neurons in the ARC; ↓protein and mRNA expression	x	x	x	Zhang et al. (2022)
Rat | Wistar	50% caloric restriction	PD 14 to LD 21	Male	PND 4, 7, 10, 14, 17, 21, and 30	↓ mRNA expression on PND 14, 17, 21, and 30; ↓ mRNA expression in the ARC on PND 21	x	x	= NPY mRNA expression	Delahaye et al. (2008)
Rat | Sprague–Dawley	50% caloric restriction	PD 10 to PD 21	Male and female	PND 21	↓ mRNA and protein expression	x	↑ MC4R mRNA and protein expression	↓NPY mRNA and protein expression	Lee et al. (2020)
Rat | Sprague–Dawley	50% caloric restriction	PD 11 to LD 21	Male and female	PND 21	↓ mRNA expression	x	x	↑ AgRP and NPY mRNA expression	Gibson et al. (2015)
Rat | Wistar	50% protein restriction	PD 0 to LD 21	Male	PND 21	x	x	x	↓ NPY neuronal number in the ARC	Plagemann, Waas, et al. (2000)
Rat | Wistar	50% protein restriction	PD 0 to LD 21	Male	PND 21	x	x	x	↑ NPY content in the PVN and LHA; no changes in NPY levels in the ARC, VMH and DMH	Plagemann, Harder, et al. (2000)
Rat | Sprague–Dawley	50% protein restriction	PD 0 to PD 21	Male	PND 21	= mRNA expression	x	x	↑ AgRP and NPY mRNA expression	Coupé et al. (2009)
Rat | Wistar	20% caloric restriction	PD 0 to 12	Male and female	PND 25	= mRNA expression	x	x	= NPY mRNA expression	Palou et al. (2012)
Rat | Wistar	20% caloric restriction	PD 1–12	Male and female	PND 25	↓mRNA expression	↓neurons in the ARC	x	↓NPY mRNA expression in females; = in AgRP mRNA expression	García et al. (2010)
Sheep	40% caloric restriction	PD 54 to birth	Female	PND 54	= neuronal number and fiber density in the ARC	x	x	= NPY and AgRP fiber density in the PVN	Prezotto et al. (2018)
Rat | Wistar	50% protein restriction	PD 6 to LD 21	Female	PND 60	↓ mRNA expression	x	x	↑ AgRP and NPY mRNA expression	Carrillo et al. (2016)
Mouse | Swiss	70% protein restriction	PD 0 to PD 21	Male	PND 112	↓mRNA expression	x	x	= NPY mRNA expression	Peixoto‐Silva et al. (2011)
Rat | Wistar	70% caloric restriction	PD 1–21	Male	PND 120	↑ mRNA expression and fiber density in PVN	x	x	= NPY fiber density or mRNA expression; ↑ beta‐endorphin fibers in PVN and DMH	Breton et al. (2009)
Rat | Wistar	70% caloric restriction	PD 0–21	Female	PND 168	= mRNA expression	x	x	↓ AgRP mRNA expression; = NPY mRNA expression	Ikenasio‐Thorpe et al. (2007)
Rat | Wistar	20% caloric restriction	PD 0–12	Male and female	PND 180	↓mRNA expression in females only	x	x	= NPY mRNA expression	Palou et al. (2012)
Rat | Sprague–Dawley	50% caloric restriction	PD 10 to PD 21	Male and female	PND 180	= mRNA expression	x	= mRNA expression	= mRNA expression	Lee et al. (2020)
Mouse | C57Bl/6J	50% protein restriction	PD 0 to LD 21	Male	PND 224	= mRNA expression	x	= MC4R mRNA expression	x	Zheng et al. (2021)
Rat | Sprague–Dawley	50% protein restriction	PD 0 to LD 21 or PD 0 to PD 21	Male	PND 240	= mRNA expression	x	x	= NPY mRNA expression	Coupé et al. (2009)

*Note:* If not specified, changes relate to the hypothalamus.

Abbreviations: —, not specified; =, no changes; ED, embryonic day; LD, lactation day; PD, pregnancy day; PND, postnatal day; x, not evaluated.

### 4.1 Maternal Undernutrition

Poor maternal nutrition has been identified as a factor that influences the metabolic outcomes of offspring in humans and other animals (Tobi et al. 2014; Victora et al. 2008; Picó et al. 2012). To study mechanisms of action, the maternal nutritional environment can be manipulated through models of global caloric restriction or macronutrient‐specific restriction, with emphasis on protein restriction (Remmers and Delemarre‐van De Waal 2011). Importantly, the use of these modes of caloric or nutrient restriction results in alterations in the development of the melanocortin system.

For instance, a 50%–70% caloric restriction during the times in which the hypothalamic nuclei important for metabolic regulation are generated (ED10‐term) results in a reduction of hypothalamic POMC mRNA expression in hypothalamic‐derived neuronal precursor cells and in the number of POMC immunoreactive neurons in the Arc of the offspring of restricted dams that persist until birth (Caminos et al. 2008; Zhang et al. 2022). Using a similar caloric restriction paradigm (50% caloric restriction), Shin et al. found decreased MC3 and MC4R mRNA expression in the hypothalamus of offspring at weaning (Shin et al. 2012). Similar results were obtained in a study where rat dams were fed 80% of the caloric intake from controls in the first 10 days of pregnancy. The offspring of these dams showed lower hypothalamic POMC mRNA expression and α‐MSH and NPY protein expression at weaning, suggesting that caloric restriction has long‐lasting consequences for the development of the melanocortin system (García et al. 2010; Palou et al. 2012). Importantly, these effects seem more predominant in females (Palou et al. 2012).

There are however some inconsistencies in the literature. For example, a study in a mouse model of 50% protein restriction has shown an increased POMC expression on ED 12 (Terroni et al. 2005). In sheep, a 20%–30% caloric restriction before and/or during pregnancy also resulted in no changes in the offspring's POMC mRNA expression (Stevens et al. 2010; Begum et al. 2012; Adam et al. 2015). These different effects may be due to the caloric restriction being mild or instead of being a restriction only in protein. Nevertheless, the effects of maternal undernutrition in AgRP/NPY neurons seem to be more consistent. AgRP and NPY mRNA expression were increased following caloric and protein restriction during the embryonic period in rats (Caminos et al. 2008), mice (Terroni et al. 2005), and sheep (Adam et al. 2015), although some studies did not report changes in those neuropeptides' levels (Terroni et al. 2005; Stevens et al. 2010). Alternatively, there may be species differences in the effects of caloric restriction during pregnancy. Indeed, a 50% maternal caloric restriction in pigs reduces hypothalamic POMC mRNA expression at birth, but only in the female offspring (Óvilo et al. 2014), with no changes in MC4R, AgRP, and NPY mRNA expression (Óvilo et al. 2014). Moreover, an increase in POMC expression may not necessarily reflect an increase in α‐MSH neurons and could instead reflect lack of enzymatic modification of POMC into α‐MSH.

As mentioned above, the effects of caloric restriction during gestation can be long‐lasting and dependent on sex. Those effects persist during postnatal development, also affecting the connections of the POMC and AgRP neurons. Up until weaning, the number of POMC neurons is still low in the ARC of both males and females born to caloric‐restricted dams (50% restriction), accompanied by lower mRNA and protein levels (Zhang et al. 2022; Delahaye et al. 2008; Gibson et al. 2015). However, those studies evaluate males and females independently, which impairs our understanding of possible sex‐specific differences. However, a 50% caloric restriction only during pregnancy also reduced POMC and NPY mRNA and protein expression at weaning in both sexes (Lee et al. 2020), while hypothalamic MC4R expression was increased (Lee et al. 2020). Importantly, a 20% reduction in maternal caloric intake in the first half of pregnancy is also sufficient to decrease POMC mRNA expression on PND 25 in male and female offspring, and NPY expression in females only, with no changes in AgRP expression (García et al. 2010). This is accompanied by fewer α‐MSH neurons in the ARC and hyperphagia (García et al. 2010). Maternal caloric restriction (50%) during pregnancy and lactation, however, led to higher AgRP and NPY mRNA levels at weaning (Delahaye et al. 2008; Gibson et al. 2015). A 50% maternal protein restriction decreases the number of NPY neurons in the ARC and α‐MSH and AgRP fibers in the PVN of male offspring at weaning (Coupé et al. 2010; Plagemann, Harder, et al. 2000; Plagemann, Waas, et al. 2000), despite increasing AgRP and NPY hypothalamic mRNA expression (Coupé et al. 2009) and specifically in the PVN and LHA (Plagemann, Harder, et al. 2000; Plagemann, Waas, et al. 2000).

When the offspring reaches adulthood, the effects of maternal caloric and protein restriction on POMC neurons are less clear and may be due to disruptions of the postnatal leptin surge. Maternal caloric restriction during pregnancy blunts the leptin neonatal surge (Palou et al. 2012; Delahaye et al. 2008), which impairs axon development from ARC POMC and AGRP/NPY neurons and leads to adulthood outcomes linked to vulnerability to obesity. Although a 20% maternal caloric restriction decreased POMC mRNA expression in female offspring (Palou et al. 2012), a 70% caloric restriction increased mRNA expression and POMC fiber density in the PVN of male offspring (Breton et al. 2009). No changes in POMC neuronal number and expression have also been reported after 50%–70% caloric restriction (Lee et al. 2020; Ikenasio‐Thorpe et al. 2007). However, the effects on AgRP/NPY seem more consistent, mostly indicating no changes in NPY mRNA expression (Palou et al. 2012; Lee et al. 2020; Breton et al. 2009; Ikenasio‐Thorpe et al. 2007), but also a decrease in hypothalamic AgRP expression (Ikenasio‐Thorpe et al. 2007). Interestingly, Breton et al. have also described an increase in beta‐endorphin fibers in the PVN of male offspring (Breton et al. 2009). The type of nutrient restriction plays an important role in the offspring outcomes, as both 50% and 70% protein restriction reduced POMC mRNA expression in adult males (Peixoto‐Silva et al. 2011) and females (Carrillo et al. 2016), although 8‐month‐old males had no differences in hypothalamic POMC and AgRP mRNA expression (Coupé et al. 2009). Adult females have also shown an increased expression of AgRP and NPY (Carrillo et al. 2016). There are, however, no studies that explore neonatal leptin levels and how they contribute to synaptic formation and axon development following maternal undernutrition.

### 4.2 Maternal Obesity

Human studies have linked maternal obesity to obesity in their children (Gaillard et al. 2014; Tie et al. 2014; Yu et al. 2013). To model this, a number of paradigms have been created where the maternal nutritional environment is manipulated by offering high‐fat or high‐sugar diets to rodent females before conception, during pregnancy, and/or lactation to replicate human observations (Remmers and Delemarre‐van De Waal 2011; Schoonejans and Ozanne 2021). These paradigms include diet manipulations to mimic exposure to unhealthy diets, like access to hypercaloric, high‐fat, and high‐sugar diets, and cafeteria diets (Tsan et al. 2021). In all these models, a variety of deleterious effects have been observed in the offspring, including hyperphagia, increased body adiposity, elevated levels of triglycerides, hyperglycemia, and insulin resistance (Menting et al. 2019).

Although our focus is on how maternal nutritional status during pregnancy and lactation may affect offspring development, most of the studies on maternal overnutrition/obesity expose dams to a high‐fat/high‐sugar diet weeks before conception and throughout pregnancy and lactation, and it is important to note that this exposure before conception can also affect the reproductive axis of the dam, including ovulation and implantation (Schoonejans and Ozanne 2021). These studies attempt to mimic the effects of maternal obesity prior to conception, but they make it difficult to determine specific vulnerability developmental windows for programming offspring vulnerability to develop obesity.

The development of the melanocortin system can be affected by HFD exposure during pregnancy or prepregnancy obesity development. Male and female offspring from dams with access to a 60%‐fat diet during pregnancy showed an increase in POMC, MC4R, and NPY mRNA expression during fetal development despite no changes in AgRP expression (Klein et al. 2018). Evaluating only males, Song et al. did not report changes in hypothalamic POMC mRNA expression but did report an increase in both AgRP and NPY expression (Song et al. 2020). Changes in MC4R expression have also been reported in male fetuses of dams with access to a 36%‐fat diet prior to mating and through pregnancy (Gout et al. 2010).

Maternal obesity induced by a 34%–60%‐fat diet before conception and maintained during pregnancy reduces or does not change POMC mRNA expression in the hypothalamus in male offspring at birth (Desai et al. 2020, 2016; Morris and Chen 2009; Lemes et al. 2018). However, AgRP expression is usually increased in these offspring (Gout et al. 2010; Desai et al. 2020, 2016; Lemes et al. 2018) despite one study describing no differences in hypothalamic AgRP mRNA expression and a decrease in NPY expression (Morris and Chen 2009). The majority of these studies have evaluated only the male offspring, but when females were also evaluated, they showed a decrease in MC4R mRNA expression similar to that observed in males, despite no differences in their POMC levels (Morris and Chen 2009), suggesting possible sex differences in the function of the melanocortin system at birth.

Most of the data suggest that a maternal 40%–70%‐fat diet through pregnancy and lactation does not change POMC mRNA expression and neuronal number in the offspring at weaning (Lemes et al. 2018; Beck et al. 2012; Gali Ramamoorthy et al. 2018; Marco et al. 2014; Vogt et al. 2014), including no changes in α‐MSH neurons and its fiber density (Lemes et al. 2018; Kirk et al. 2009), although those studies evaluated males or females independently. Despite no changes in mRNA expression itself, the POMC promoter region has been shown to be hypermethylated (Marco et al. 2014), which could affect its transcription throughout life. However, diets with a lower fat content (around 35%) led to an increase in POMC mRNA expression in weaning males (Gout et al. 2010; Chen and Morris 2009; Chen et al. 2008), suggesting that the percentage of fat might be a critical factor during the development of POMC neurons. Furthermore, it has also been reported a decrease in POMC and α‐MSH fiber density in the PVN, DMH, and LHA, with no changes in total neuronal number in the ARC (Vogt et al. 2014; Park et al. 2020), and a decrease in hypothalamic POMC mRNA expression (Nguyen et al. 2017). The MC4R expression has been evaluated only in males, where a maternal 34% or 60%‐fat diet did not induce changes in expression at weaning (Gali Ramamoorthy et al. 2018; Chen and Morris 2009; Chen et al. 2008) or decrease its expression in the hypothalamus (Nguyen et al. 2017).

Inversely, AgRP/NPY neurons seem to be affected at weaning by different models of maternal obesity. Exposure to a HFD (34%–60% kcal from fat) decreases hypothalamic and ARC AgRP and NPY mRNA expression in male offspring (Gout et al. 2010; Gali Ramamoorthy et al. 2018; Chen et al. 2008), and also a decrease in AgRP fiber density in the PVN, DMH, and LHA (Vogt et al. 2014; Kirk et al. 2009). However, the mRNA expression of these neuropeptides and their neuronal numbers have not changed following exposure to similar diets (34%–70% kcal from fat) (Lemes et al. 2018; Beck et al. 2012; Vogt et al. 2014; Chen and Morris 2009; Park et al. 2020). These studies, however, evaluated mostly males, limiting our understanding of the melanocortin system at weaning in both sexes. For example, when evaluating only the female offspring, Nguyen et al. found a significant increase in NPY mRNA expression at weaning (Nguyen et al. 2017), which has not been reported in males, apart from an increase in NPY neuronal number in ARC on PND 28 (Lemes et al. 2018).

Impairments in the melanocortin system due to maternal obesity/HFD exposure extend until adulthood. Frequently, POMC mRNA expression and neurons in the ARC are decreased following maternal HFD intake (from 34%–60% kcal from fat) (Desai et al. 2020, 2016; Chen et al. 2008; Park et al. 2020; Couvreur et al. 2011; Ornellas et al. 2016; Cleal et al. 2019; Dearden et al. 2020; Li et al. 2021), which is also accompanied by a decrease in α‐MSH fiber density in the PVN, DMH, and LHA and protein expression (Vogt et al. 2014; Ornellas et al. 2016). Unfortunately, most of these results were observed only in the male offspring, as few have analyzed both males and females. Couvreur et al., however, have shown that only males show a decreased hypothalamic POMC mRNA expression at 23 weeks of age, while females were not affected by the maternal cafeteria diet (Couvreur et al. 2011). This lack of change in POMC neurons in females has also been observed in other studies that evaluated only female offspring (Marco et al. 2014; Rajia et al. 2013) or both males and females (Kulhanek et al. 2020; Schellong et al. 2020). The MC4R mRNA expression has not been extensively studied, but it seems not to be affected by maternal obesity in adult offspring following a 60%‐fat diet exposure (Gali Ramamoorthy et al. 2018; Kulhanek et al. 2020; Gawliński et al. 2020). However, male offspring from dams with access to a cafeteria diet (30% kcal from fat) had an increased MC4R mRNA expression in the posterior hypothalamus (Chen et al. 2009). Interestingly, a maternal high‐sugar diet exposure also increased MC4R expression in the ventral tegmental area and nucleus accumbens of male offspring (Gawlinska et al. 2020), suggesting that the impacts of maternal obesity in offspring energy balance might be seen in other brain areas related to food intake control or modulation.

While POMC neurons are frequently reduced or unaltered following maternal obesity, the effects on AgRP/NPY neurons are less consistent and not necessarily related to the changes observed in POMC neurons. A maternal 45%–60%‐fat diet intake increased both AgRP and NPY hypothalamic mRNA expression in adult offspring (Desai et al. 2020, 2016; Ornellas et al. 2016; Dearden et al. 2020; Li et al. 2021), despite only one of those studies examining both males and females, with no sex differences in mRNA expression (Ornellas et al. 2016). Although this upregulated expression is associated with a downregulated POMC expression in these previous studies, it has also been reported a global POMC and AgRP/NPY downregulation or a decrease in AgRP/NPY mRNA expression and fiber density independent of any changes in POMC neurons (Vogt et al. 2014; Park et al. 2020; Couvreur et al. 2011; Cleal et al. 2019; Schellong et al. 2020; Chen et al. 2009, 2014). In addition, the POMC promoter region was hypermethylated in both male and female offspring independently of a decreased mRNA expression (Gali Ramamoorthy et al. 2018; Schellong et al. 2020), while AgRP hypermethylation was observed only in males (Schellong et al. 2020), demonstrating that changes in transcription could account for changes in the melanocortin system and should be further evaluated. These data indicate that, in adulthood, maternal obesity may induce changes that favor the orexigenic pathway, through the upregulation of AgRP/NPY neurons and downregulation of POMC neurons. Inversely, it may also promote a global downregulation of the melanocortin system, which could be related to widespread effects in brain development, like reduced neuronal number.

The period of exposure and diet composition could partially explain some of the different outcomes seen in the offspring, as offspring age and sex, which impair the comparison between studies and the determination of the most probable offspring outcome, if any. For instance, a couple of studies in which dams were exposed to diets containing between 40% and 70% of fat have not reported any effects of maternal HFD intake on POMC and AgRP/NPY neurons (Beck et al. 2012; Gali Ramamoorthy et al. 2018; Rajia et al. 2013; Kulhanek et al. 2020).

### 4.3 Maternal Hyperglycemia and Insulin Resistance

Maternal hyperglycemia increases the risk of the offspring developing obesity, insulin resistance, and metabolic syndrome (Lawlor et al. 2011; Catalano and deMouzon 2015; Perng et al. 2020; Hillier et al. 2007). In animal models, maternal hyperglycemia can be induced through energy‐dense diets that lead to obesity or via hypoinsulinemia, which is especially important to determine the effects of maternal hyperglycemia independent of obesity (Kleinert et al. 2018). The administration of β‐cytotoxic agents such as streptozotocin (STZ) or alloxan decreases maternal glucose clearance and increases glucose transport to the fetus. Importantly, maternal hyperglycemia alters the development of offspring hypothalamic circuits involved in food intake control (Plagemann et al. 1998; Franke et al. 2005; Steculorum and Bouret 2011; Martins et al. 2023; Fahrenkrog et al. 2004).

At weaning, maternal hyperglycemia increased the number of NPY neurons in the offspring's ARC (Plagemann et al. 1998), while females showed no differences in the number of POMC, α‐MSH, AgRP, and NPY neurons (Franke et al. 2005). Males born to hyperglycemic dams showed decreased AgRP and α‐MSH projections to the PVN, with an increased number of α‐MSH neurons in the ARC in adulthood (Steculorum and Bouret 2011), which are accompanied by hyperphagia, hyperglycemia, and obesity (Steculorum and Bouret 2011). A recent study from our group showed that maternal hyperglycemia during pregnancy affects offspring POMC expression in a sex‐dependent manner (Martins et al. 2023). Females born to mildly hyperglycemic dams had fewer POMC neurons on ED 19, while males showed no differences. In adulthood, however, the number of POMC neurons in the ARC increased in both males and females (Martins et al. 2023). Interestingly, exposure to maternal hyperglycemia only during lactation (i.e., through maternal milk) was sufficient to decrease both POMC and α‐MSH neurons in the ARC, with no changes in NPY neuronal number (Fahrenkrog et al. 2004). Although the data are scarce and with no proper evaluation of the development of the melanocortin system in male and female offspring, it seems that maternal hyperglycemia results in changes in the balance of the melanocortin system that are biased to promote food intake and weight gain, which could explain the vulnerability of these offspring developing obesity.

### 4.4 Maternal Exposure to Endocrine Disruptors

Most recently, maternal exposure to different endocrine disruptors has also played a role in fetal programming, as it has been correlated to the development of obesity in the offspring. Prenatal exposure to different endocrine disruptors, such as dichlorodiphenyldichloroethylene (DDE), hexachlorobenzene (HCB), and polychlorinated biphenyls (PCBs), widely used pesticides, have been associated with increased adiposity, BMI, and cardiovascular effects in children (Tang‐Péronard et al. 2014; Vafeiadi et al. 2015; Verhulst et al. 2009). Similarly, pre and postnatal exposure to tobacco smoking is associated with overweight in young children (Møller et al. 2014; Rönn et al. 2014).

Despite possible mechanisms related to adipocyte function (Darbre 2017), impairments in the melanocortin system may also contribute to the obesogenic phenotype seen in the offspring, and a couple of animal model studies have evaluated those potential mechanisms. Although an acute exposure to polychlorinated biphenyl (PCB) mixture at the end of pregnancy (DP 16 and 18) was not sufficient to alter MC3R mRNA expression in adult male and female offspring (Gore et al. 2021), chronic exposure during the fetal and postnatal development is. Exposure to bisphenol‐A (BPA, 0.02 ppm) during pregnancy and lactation decreased POMC fiber density in the PVN in both male and female mice at weaning and adulthood, without changing the number of cell bodies in the ARC or the AgRP and NPY mRNA expression (Mackay et al. 2013, 2017). Moreover, BPA exposure delayed the postnatal leptin surge to disrupt the development of the melanocortin projections from the ARC to the PVN (MacKay et al. 2017), indicating that endocrine disruptors may affect the secretion of hormones that modulate the development of the melanocortin system.

### 4.5 Nonhuman Primate Models

The developmental trajectories of the melanocortin system in nonhuman primates differ from that observed in rodents, but it could be a better model for human hypothalamic development. The majority of hypothalamic AgRP/NPY projections are established before birth (Grayson et al. 2006), although their level is not the same as seen in adulthood. These data suggest that in nonhuman primates and, possibly, humans, maternal metabolic impairments during pregnancy alone might be more critical to determining the development of both neurons and their projections in the offspring, whereas in rodents, lactation has a greater contribution to the development of the melanocortin pathways. Nonetheless, offspring outcomes following maternal metabolic impairments have been less extensively studied in primates.

Available studies show that a 70% caloric restriction in baboons during pregnancy decreased the number of POMC neurons in the ARC, while increasing NPY neurons in male and female offspring at birth (Li et al. 2013). A nonhuman primate model (*Macaca fusata*) of maternal obesity induced by a 30%‐fat diet showed an increased expression of POMC and MC4R mRNA in the ARC of both male and female offspring during fetal development, accompanied by a decrease in AgRP hypothalamic mRNA expression and fiber density in the PVN (Grayson et al. 2010). Adult offspring also showed a decreased AgRP fiber density in the ARC and the PVN, despite no changes in α‐MSH fiber density in the PVN (Sullivan et al. 2017). Interestingly, exposure to an HFD only prior to pregnancy did not change POMC and AgRP expression in nonhuman primate fetuses (Grayson et al. 2010), indicating that an increased intake of fat during fetal development might be more detrimental than maternal body weight per se. Furthermore, this suggests that diet management during pregnancy could be a way of promoting better outcomes for the offspring, regardless of maternal preconception nutritional status.

As described previously, exposure to endocrine disruptors, such as nicotine, is correlated to the development of obesity during childhood. A study evaluating nicotine exposure throughout pregnancy in nonhuman primates showed that, at birth, there is an increase in POMC mRNA expression in the ARC and a decrease in NPY expression in the offspring (Grove et al. 2001). Although these data indicate changes related to promoting food intake inhibition, this offspring also had a remarkable reduction in leptin levels (Grove et al. 2001), suggesting that changes in leptin signaling and the melanocortin system postnatally could be related to an increased risk of developing obesity. Differently from what is seen in rodents, there is no evidence that the development of ARC projections is dependent on a leptin surge in nonhuman primates (Grayson et al. 2006). In addition to changes in leptin signaling, nicotine exposure may also alter cholinergic inputs to the melanocortin system since nicotinic receptors' activation in POMC neurons of the ARC increases their firing rate and induces hypophagia (Mineur et al. 2011).

## 5 General Remarks

**FIGURE 2:**
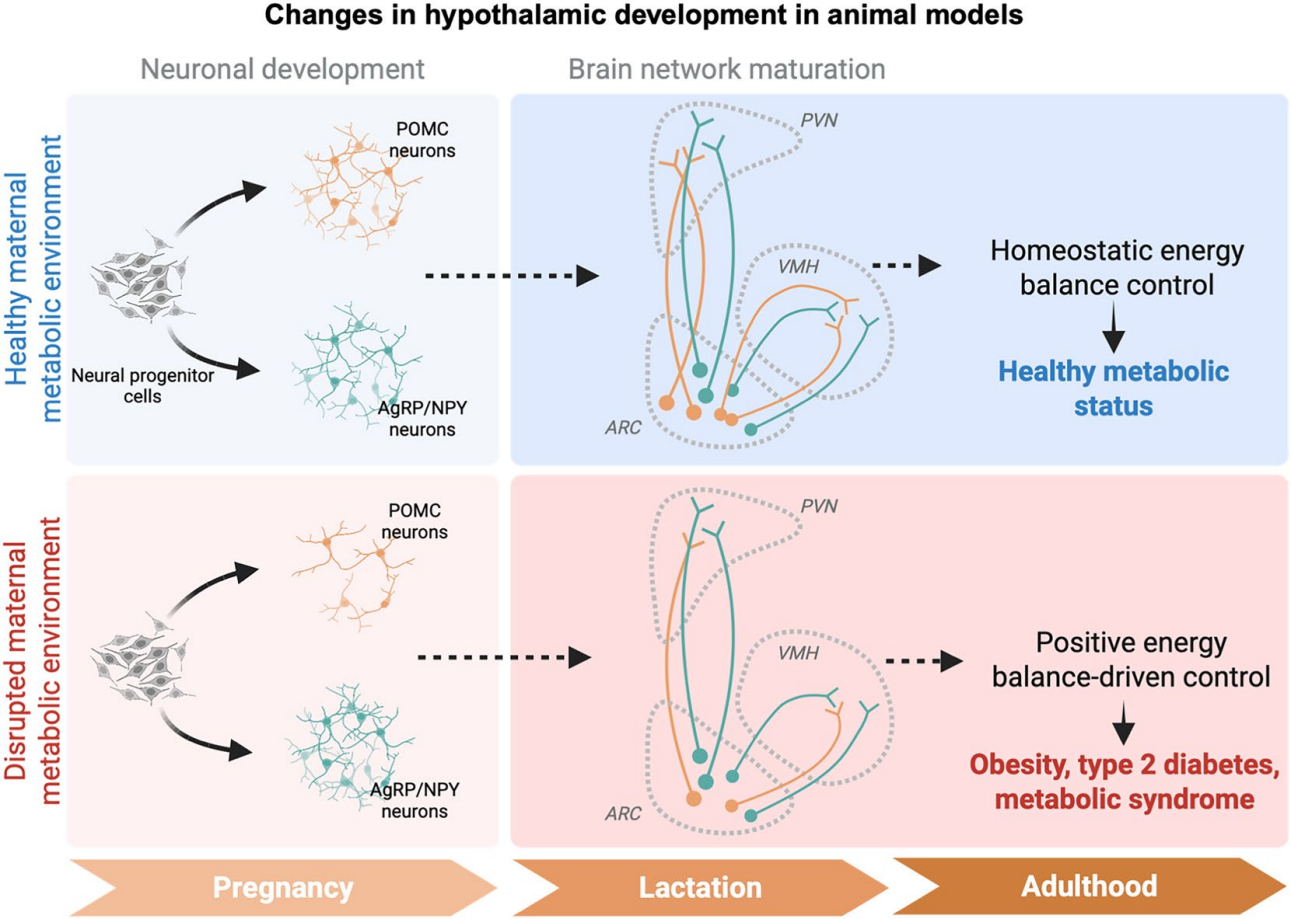
Changes in hypothalamic development in animal models. Preclinical animal studies suggest that changes in the development of the melanocortin system may be one of the key mechanisms underlying the adult vulnerability to develop energy balance alterations which ultimately would lead to obesity in offspring raised by mothers that were energetically challenged during pregnancy and lactation (Created in BioRender, https://BioRender.com/71h2bvr).

Maternal metabolic impairments are related to an increased risk of obesity in the offspring throughout life, and changes in the development of the melanocortin system may be one of the mechanisms that promote increased food intake in the long term, leading to a positive energy balance and increased fat deposition (see Figure 2).

One of the most common findings in this review is that reduced nutrient availability during pregnancy and lactation impacts primarily ARC POMC mRNA expression and the development of ARC POMC projections to target sites within the hypothalamus. The impact of poor nutrition on AgRP/NPY neurons seemed to be less consistent. These data suggest that, following maternal undernutrition, the development of obesity in the offspring might be more related to a decrease in POMC neuronal function, which could result in increased food intake, reduced metabolic rate, and altered glucose homeostasis, ultimately making these offspring more vulnerable to develop metabolic disorders.

A more complex scenario is revealed when the maternal environment is one of overnutrition. Indeed, maternal obesity animal models generally result in an upregulation in late gestational and early postnatal expression of AgRP/NPY mRNA and a downregulation of POMC mRNA in the ARC. This pattern of results, however, became less consistent in studies where AgRP/NPY mRNA expression or fiber density were examined in adult animals, suggesting that there are compensatory mechanisms that correct the impact of high‐calorie diets on the development of these projections. Alternatively, the lack of a consistent pattern in these results could be due to the varied number of dietary manipulations (i.e., exposure to a 45% vs. 60% high‐fat diet), the length and timing of the exposures to these manipulations (i.e., exposure during gestation only, lactation only or gestation and lactation), the type of diet provided (i.e., cafeteria diet vs. high‐fat diet), the age at which the offspring were sacrificed and the strain of rats or mice used.

It is important to highlight that the exposure timeline is crucial to determine the nature of the impact expected, knowing the normal patterns of hypothalamic development, which could lead to substantially different outcomes in the offspring (Remmers and Delemarre‐van De Waal 2011). Both the quantity and the quality of nutrients could affect offspring development differently and therefore explain the multiple effects seen in the melanocortin system. The impact of under‐ or over‐nutrition during pregnancy on programming offspring metabolism is potentially modulated by the placental barrier. Nevertheless, continued exposure to high levels of fatty acids and/or glucose can cause a chronic increase in inflammatory signals that compromise the placental barrier (Dearden and Ozanne 2015). Ultimately, this would impact fetal hypothalamic development, as would be reflected in disrupted expression of key transcription factors that regulate gene expression in ARC POMC and AgRP/NPY precursors and ultimately change their developmental fate (Cone 2005; Padilla et al. 2010; MacKay and Abizaid 2014; Lu et al. 2013). Lactation is also a key period to the offspring's development, as changes in the maternal nutritional environment are conveyed to the offspring through nursing. Moreover, the projections from melanocortin peptidergic cells in the ARC to their targets in the hypothalamus and other regions occur after birth in mice and rats, and thus these may be sensitive to changes in maternal diets during the lactation period. Besides its nutritional value, breast milk delivers hormones, growth factors, microbiota, and milk‐derived extracellular vesicles (MEVs) to the offspring (Wijenayake et al. 2023), which contribute to offspring development (Eisha et al. 2022). Specifically, the impact of maternally transmitted MEVs is an emerging and exciting field of study indicating a path through which mothers continue to modulate brain development during the postnatal period (Jiang et al. 2021), as they have been linked to neuronal differentiation and synaptic maturation (Jiang et al. 2021; Bian et al. 2013) and could potentially mediate the melanocortin system development.

Other factors that affect offspring outcomes include the age and sex of animals being studied (Remmers and Delemarre‐van De Waal 2011; Picó et al. 2012). From studies reviewed, most had adult endpoints that included young animals (most were between the ages of 2–4 months with one study looking at 5‐month‐old rodents; see Tables 1 and 2). While metabolic disorders like obesity and type II diabetes are becoming more prevalent in young individuals in human populations, the impact of these disorders is clearer in mid to late adulthood and into aging. Thus, the impact of maternal dietary challenges or impaired metabolic function on the offspring may be more evident if the offspring are examined at later developmental time points (Schoonejans and Ozanne 2021; Rodríguez‐González et al. 2019).

**TABLE 2:** Animal models of maternal obesity and the development of the melanocortin system.

Species	Maternal intervention	Duration	Offspring	Age	Outcomes	a‐MSH	Receptors	AgRP/NPY	References
	Diet composition		Sex		POMC				
Rat | Wistar	HFD (60.3% kcal from fat)	PD 0 to PD 18	Male and female	ED 12, 14, 16, and 18	↑ mRNA expression on E18	x	↑ MC4R mRNA expression on E12	↑ NPY mRNA expression on E18; = AgRP mRNA expression	Klein et al. (2018)
Mouse | C57BL/6J	HFD (36.1% kcal from fat)	PD −35 to LD 21	Male	ED 19.5	x	x	↑ MC4R mRNA expression	x	Gout et al. (2010)
Rat | Sprague–Dawley	HFD (60% kcal from fat)	PD 0–21	Male	ED 20	= mRNA expression	x	x	↑ AgRP and NPY mRNA expression	Song et al. (2020)
*Macaca fusata*	HFD (30% kcal from fat)	2–5 years prior mating to c‐section	Male and female	ED 130	↑ mRNA expression in the ARC	= fiber density in the PVN	↑ MC4R mRNA expression	↓ AgRP mRNA expression; ↓ AgRP fiber density in the PVN	Grayson et al. (2010)
Rat | Sprague–Dawley	Cafeteria diet (34% from fat)	PD −35 to PD 21	Male and female	PND 1	↓ mRNA expression only in males	x	↓ MC4R mRNA expression in males and females	↓ NPY mRNA expression in both sexes; = in AgRP mRNA expession	Morris and Chen (2009)
Rat | Sprague–Dawley	HFD (60% kcal from fat)	PND 21 to LD 21	Male	PND1	= mRNA expression	x	x	↑ AgRP mRNA expression	Desai et al. (2016)
Mouse | C57BL/6J	HFD (45% kcal from fat)	PND 21 to LD 21	Male	PND1	↓ mRNA expression	x	x	↑ AgRP mRNA expression	Desai et al. (2020)
Mouse | Swiss	HFD (46% kcal from fat)	PD −21 to LD 21	Male	PND 1	= mRNA expression	x	x	↑ AgRP mRNA expression	Lemes et al. (2018)
Mouse | C57BL/6J	HFD (36.1% kcal from fat)	PD −35 to LD 21	Male	PND 1	x	x	↑ MC4R mRNA expression	↑ AgRP mRNA expression	Gout et al. (2010)
Mouse | C57BL/6J	HFD (58% kcal from fat)	PD −42 to LD 21	Male and female	PND 10	↓ fiber density in the PVN; = neuronal number in the ARC	↓ fiber density in the PVN	x	= AgRP neuronal number in the ARC and fiber density in the PVN	Park et al. (2020)
Mouse | C57BL/6J	HFD (36.1% kcal from fat)	PD −35 to LD 21	Male	PND 10 and 21	↑ mRNA expression	x	x	↓ AgRP mRNA expression	Gout et al. (2010)
Rat | Sprague–Dawley	Cafeteria diet (34% kcal from fat)	PD −35 to LD 21	Male	PND 19	↑ mRNA expression	x	= MC4R mRNA expression	= NPY and AgRP mRNA expression	Chen and Morris (2009)
Rat | Sprague–Dawley	Cafeteria diet (34% kcal from fat)	PD −35 to LD 20	Male	PND 20	↑ mRNA expression	x	= MC4R mRNA expression	↓ NPY mRNA expression	Chen et al. (2008)
Rat | Sprague–Dawley	HFD (43.5% kcal from fat)	PD −42 to LD 21	Female	PND 21	↓ mRNA expression	x	↓ MC4R mRNA expression	↑ NPY mRNA expression	Nguyen et al. (2017)
Rat | Sprague–Dawley	HFD (60% kcal from fat)	PD −42 to LD 21	Male	PND 21	= mRNA expression in the ARC	x	= MC4R mRNA expression in the PVN	↓ AgRP and NPY mRNA expression in the ARC	Gali Ramamoorthy et al. (2018)
Rat | Long‐Evans	HFD (70% kcal from fat) | HC (70% kcal from carbs)	PD 12–21	Male	PND 21	= mRNA expression in the ARC	x	x	= NPY mRNA expression in the ARC	Beck et al. (2012)
Rat | Sprague–Dawley	HFD (60% kcal from fat)	PD 0 to LD 21	Female	PND 21	= protein expression	x	x	= NPY protein expression	Song et al. (2020)
Mouse | C57BL/6J	HFD (55.2% kcal from fat)	LD 0–21	Male	PND 21	= mRNA expression and neuronal number in the ARC; = eletrophysiological properties	↓ fiber density in the PVN, DMH, and LHA	x	=AgRP and NPY mRNA expression in the ARC; = AgRP neuronal number in the ARC; ↓ AgRP fiber density in the PVN, DMH, and LHA	Vogt et al. (2014)
Rat | Wistar	HFD (60% kcal from fat)	PND 21 to LD 21	Female	PND 22	= mRNA expression	x	x	x	Marco et al. (2014)
Mouse | Swiss	HFD (46% kcal from fat)	PD −21 to LD 21	Male	PND 28	= mRNA expression	= neuronal number in the ARC	x	= AgRP mRNA expression; ↑ NPY neuronal number in the ARC	Lemes et al. (2018)
Rat | Sprague–Dawley	HFD	PD −42 to LD 21	Male and female	PND 30	x	= fiber density	x	↓ AgRP fiber density in the PVN	Kirk et al. (2009)
Mouse | C57BL/6J	High‐fat high‐sugar diet	PD −28 to LD 21	Male and female	PND 42	= mRNA expression	x	= MC4R mRNA expression	= NPY mRNA expression	Kulhanek et al. (2020)
Mouse | C57BL/6J	HFD (45% kcal from fat)	PD −42 to LD 21	Male	PND 56	↓ neuronal number and mRNA expression in the ARC	x	x	↑ NPY mRNA expression in the ARC	Dearden et al. (2020)
Rat | Sprague–Dawley	HFD (43% kcal from fat)	PD −42 to LD 21	Male	PND 63	= mRNA expression in the ARC	x	x	↓ NPY mRNA expression in the ARC	Chen et al. (2014)
Rat | Wistar	High‐sugar diet	PD 0 to LD 21	Male	PND 63	x	x	↑ MC4R levels in Nac and VTA; = in the hypothalamus	x	Gawlinska et al. (2020)
Mouse | C57BL/6J	HFD (58% kcal from fat)	PD −42 to LD 21	Male and female	PND 70	↓ fiber density in the PVN	x	x	↓ AgRP fiber density in the PVN	Park et al. (2020)
Rat | Wistar	HFD (60% kcal from fat)	PND 21 to LD 21	Female	PND 80 and 110	= mRNA expression	x	x	x	Marco et al. (2014)
Mouse | C57BL/6J	HFD (49% kcal from fat)	PD −56 to LD 21	Male and female	PND 90	↓ mRNA and protein expression	↓ protein expression	x	↑ NPY mRNA and protein expression	Ornellas et al. (2016)
Rat | Sprague–Dawley	HFD (40.7% kcal from fat)	PD −35 to LD 21	Female	PND 98	= mRNA expression in the ARC	x	x	= NPY mRNA expression in the ARC	Rajia et al. (2013)
Mouse | C57BL/6J	HFD (45% kcal from fat)	PD −56 to LD 21	Male	PND 105	↓ mRNA expression in the ARC	x	x	↓ NPY mRNA expression in the ARC	Cleal et al. (2019)
Rat | Sprague–Dawley	HFD (60% kcal from fat)	PD 0 to LD 21	Female	PND 105	↑ protein expression	x	x	= NPY protein expression	Song et al. (2020)
Mouse | C57BL/6J	HFD (60% kcal from fat)	PD 0–21	Male	PND 112	↓ mRNA and protein expression; ↓ synaptic density in the ARC	x	x	↑ NPY mRNA and protein expression; ↑ NPY synaptic density in the Arc	Li et al. (2021)
Rat | Sprague–Dawley	Cafeteria diet (34% from fat)	PD −35 to LD 21	Male	PND 126	↓ mRNA expression in the ARC	x	↑ MC4R mRNA expression in the posterior hypothalamus	↓ AgRP mRNA expression in the ARC; = in NPY mRNA expression in the ARC	Chen et al. (2009)
Mouse | C57BL/6J	HFD (55.2% kcal from fat)	LD 0–21	Male	PND 140	= mRNA expression na neuronal number in the ARC; = in eletrophysiological properties	↓ fiber density in the PVN, DMH, and LHA	x	= AgRP and NPY mRNA expression in the ARC; =AgRP neuronal number in the ARC; ↓ AgRP fiber density in the PVN, DMH, and LHA	Vogt et al. (2014)
Rat | Sprague–Dawley	HFD (60% kcal from fat)	PD −15 to LD 21	Male	PND 140	= mRNA expression in the ARC	x	= MC4R mRNA expression in the PVN	= AgRP and NPY mRNA expression in the ARC	Gali Ramamoorthy et al. (2018)
Rat | Long‐Evans	HFD (70% kcal from fat) | HC (70% kcal from carbs)	PD 12–21	Male	PND 140	= mRNA expression in the ARC	x	x	= NPY mRNA expression in the ARC	Beck et al. (2012)
Rat | Wistar	Cafeteria diet	PD −42 to LD 21	Male and female	PND 161	↓ mRNA expression in males only	x	x	↓ NPY mRNA expression in males only	Couvreur et al. (2011)
Rat | Sprague–Dawley	HFD (60% kcal from fat)	PND 21 to LD 21	Male	PND 180	↓ mRNA expression	x	x	↑ AgRP mRNA expression	Desai et al. (2016)
Rat | Wistar	HFD (34% kcal from fat)	PD −42 to LD 21	Male and female	PND 200	= mRNA expression in the ARC	x	x	↓ AgRP mRNA expresssion in the ARC in males only	Schellong et al. (2020)
Mouse | C57BL/6J	HFD (45% kcal from fat)	PNS 21 to LD 21	Male	PND 360	↓ mRNA expression	x	x	↑ AgRP mRNA expression; ↑ NPY neuronal number in the ARC	Desai et al. (2020)
*Macaca fusata*	HFD (37% kcal from fat)	2–9 years prior mating to weaning	Male and female	PND 390	x	= fiber density in the ARC and PVN	x	↓ AgRP fiber density in the ARC and PVN	Sullivan et al. (2017)

*Note:* If not specified, changes relate to the hypothalamus.

Abbreviations: =, no changes; ED, embryonic day; LD, lactation day; PD, pregnancy day; PND, postnatal day; x, not evaluated.

Another important limitation of the data available is that many studies commonly examine outcomes in males or females, which impairs the evaluation of possible sex differences. The importance of this issue is evident in the data where sex is included as a variable, showing sex differences in response to maternal diet or environmental exposure to endocrine disrupters on the development of the melanocortin system (Gibson et al. 2015; Lee et al. 2020; Martins et al. 2023; MacKay et al. 2017). Importantly, when female offspring are exposed to an impaired maternal nutritional environment during development, they have not only an increased risk of developing obesity and gestational diabetes later in life, but they also show epigenetic modifications that could perpetuate the risk of metabolic disorders through a transgenerational effect (Koletzko et al. 2014). Particularly, the melanocortin system itself shows sex‐specific differences, with males having fewer POMC neurons than females (Nohara et al. 2011), which could contribute to their hyperphagia. This suggests that there are sex differences driven by either genetic (i.e., sex chromosome related) or sex hormone dependent changes in the melanocortin system that could also interact with maternal derived factors to impact the programming of the melanocortin system. Therefore, future studies should evaluate hypothalamic development and energy balance control in both male and female offspring, taking into account sex related developmental factors including changes in circulating sex‐determining hormones (Nohara et al. 2011).

Finally, another issue rarely evaluated is the issue of compensation, where the impact of gestational and postnatal maternal factors is mitigated by compensatory mechanisms that allow affected offspring to maintain a normal phenotype, but that makes them vulnerable in the presence of a metabolic challenge in adulthood. For instance, offspring of rat dams exposed to BPA or to maternal hyperglycemia show much more severe metabolic alterations when challenged with a high‐fat diet as adults than when exposed to a lower‐calorie diet (Martins et al. 2023; MacKay et al. 2017). Thus, just like unmasking homeostatic impairments by using older animals, the use of high‐calorie diets, fasting, stress, and other metabolic challenges may be useful in better understanding compensatory processes in organisms that are exposed to nutritional imbalance early in life.

## Conflicts of Interest

The authors declare no conflicts of interest.

## Data Availability

The authors have nothing to report.
